# Synthesis, antibiotic structure–activity relationships, and cellulose dissolution studies of new room-temperature ionic liquids derived from lignin

**DOI:** 10.1186/s13068-021-01898-x

**Published:** 2021-02-23

**Authors:** Shihong Liu, Michael Gonzalez, Celine Kong, Scott Weir, Aaron M. Socha

**Affiliations:** 1 Department of Chemistry and Environmental Science, Queens University of Charlotte, 1900 Selwyn Avenue, Charlotte, NC 28274 USA; 2 Biological Systems and Engineering Division, Lawrence Berkeley National Laboratory, Berkeley, CA USA; 3 Department of Biology, Queens University of Charlotte, 1900 Selwyn Avenue, Charlotte, NC 28274 USA

## Abstract

**Background:**

Ionic liquids (ILs) are promising pretreatment solvents for lignocellulosic biomass, but are largely prepared from petroleum precursors. Benzaldehydes from depolymerized lignin, such as vanillin, syringaldehyde, and 4-methoxy benzaldehyde, represent renewable feedstocks for the synthesis of ionic liquids. We herein report syntheses of novel lignin-derived ionic liquids, with extended *N*-alkyl chains, and examine their melting points, cellulose dissolution capacities, and toxicity profiles against *Daphnia magna* and *E. coli* strain 1A1. The latter organism has been engineered to produce isoprenol, a drop-in biofuel and precursor for commodity chemicals.

**Results:**

The new *N,N*-diethyl and *N,N*-dipropyl methyl benzylammonium ILs were liquids at room temperature, showing 75–100 °C decreased melting points as compared to their *N,N,N*-trimethyl benzylammonium analog. Extension of *N*-alkyl chains also increased antibacterial activity threefold, while ionic liquids prepared from vanillin showed 2- to 4-fold lower toxicity as compared to those prepared from syringaldehyde and 4-methoxybenzaldehyde. The trend of antibacterial activity for anions of lignin-derived ILs was found to be methanesulfonate < acetate < hydroxide. Microcrystalline cellulose dissolution, from 2 to 4 wt% after 20 min at 100 °C, was observed in all new ILs using light microscopy and IR spectroscopy.

**Conclusions:**

Ionic liquids prepared from H-, S- and G-lignin oxidation products provided differential cytotoxic activity against *E. coli* and *D. magna*, suggesting these compounds could be tailored for application specificity within a biorefinery.

## Background

Ionic liquids (ILs) are salts with melting points below 100 °C, super-solvents comprised entirely of paired ions. The physical properties of ILs, e.g., viscosity, conductivity, vapor pressure and thermal stability, are defined by structural features [1], and have been exploited in over 50 commercial applications [2]. Chemical “tuning” of ionic liquids has been described [3], exploited to solvate a wide range of natural products [4], and extended to protein stabilization [5] and enzyme catalysis [6]. Depending on their degree of basicity, ILs can dissolve cellulose [7], hemicellulose [8] and/or lignin [9], effectively pretreating plant biomass for enzymatic hydrolysis to monomeric sugars [10, 11]. When compared to other biomass pretreatment methods, ILs typically provide higher space–time yields of glucose and xylose [12, 13], and fewer inhibitors of downstream fermentation [14]. Bokinski et al. [15] demonstrated microbial production of butanol, fatty acid ester, and terpene biofuels from switchgrass pretreated with 1-ethyl-3-methyl imidazolium acetate (**1**). Integrated pretreatment and fermentation unit operations, using bio-based ILs, e.g., from amino acids [16, 17], have been demonstrated, as well as aqueous IL pretreatment systems using methanesulfonate anions [18]. Applications of ILs for the production of consumer products, such as textiles [19], and foods [20] have also garnered attention for bio-based ILs [21].

Mechanistic studies show IL toxicity generally increases with cation alkyl chain length as a result of cell membrane destabilization [22, 23]. *N*-alkylated pyridinium, imidazolium and quaternary ammonium cations showed increased toxicity across trophic levels, including studies with *C. elegans, D. magna*, and mammalian cell cultures [24–30]. Degree of functionalization of cation side chains [31], localization of heteroatoms on the cation and anion [28], cation and anion chain length [32, 33], and/or ion coordination [34] can all influence bioactivity. Introduction of polar functional groups into shorter alkyl chains reduced the aquatic toxicity of a broad range of cation classes [35].

Quaternary ammonium ILs have diverse engineering applications [36], and have been listed as suitable COVID-19 disinfectants for hard, non-porous surfaces [37]. Our research focuses on biomass applications using benzylammonium ionic liquids and deep eutectic solvents synthesized from lignin depolymerization products, such as benzaldehydes [38–40], and we have recently demonstrated isolation techniques for aminophenol IL precursors from oxidized kraft lignin [41]. Diez et al. prepared a series of trimethyl benzylammonium acetate ILs (**6**–**8**) from 4-methoxy benzaldehyde, syringaldehyde, and vanillin, respectively, representing major oxidative depolymerization products from H-, S- and G-type lignin [39]. Though compounds **6**–**8** remained solid at room temperature, their utility as solvents for switchgrass pretreatment were comparable to **1**. In the present study, modification of *N*-alkyl chains using diethyl and dipropyl amines provided room-temperature ILs **9**–**10**. Cellulose solubility of the new compounds was examined using light microscopy and confirmed with IR spectroscopy.

In efforts to inform in planta lignin engineering of biorefinery feedstocks [42], the toxicity of the lignin-derived ILs were determined using an *E. coli* strain recently optimized for the production of isoprenol (3-methyl-3-buten-1-ol) [43]. As commercial production volumes of ILs increase, there is a parallel risk of a leak or spill contaminating the environment, e.g., groundwater near a biorefinery. Therefore, selected ILs (**11** and **12**) were evaluated using *Daphnia magna*, a model organism used by the United States Environmental Protection Agency and the Organization for Economic Cooperation and Development to determine chemical contamination to freshwater environments [44, 45].

**Fig. 1:**
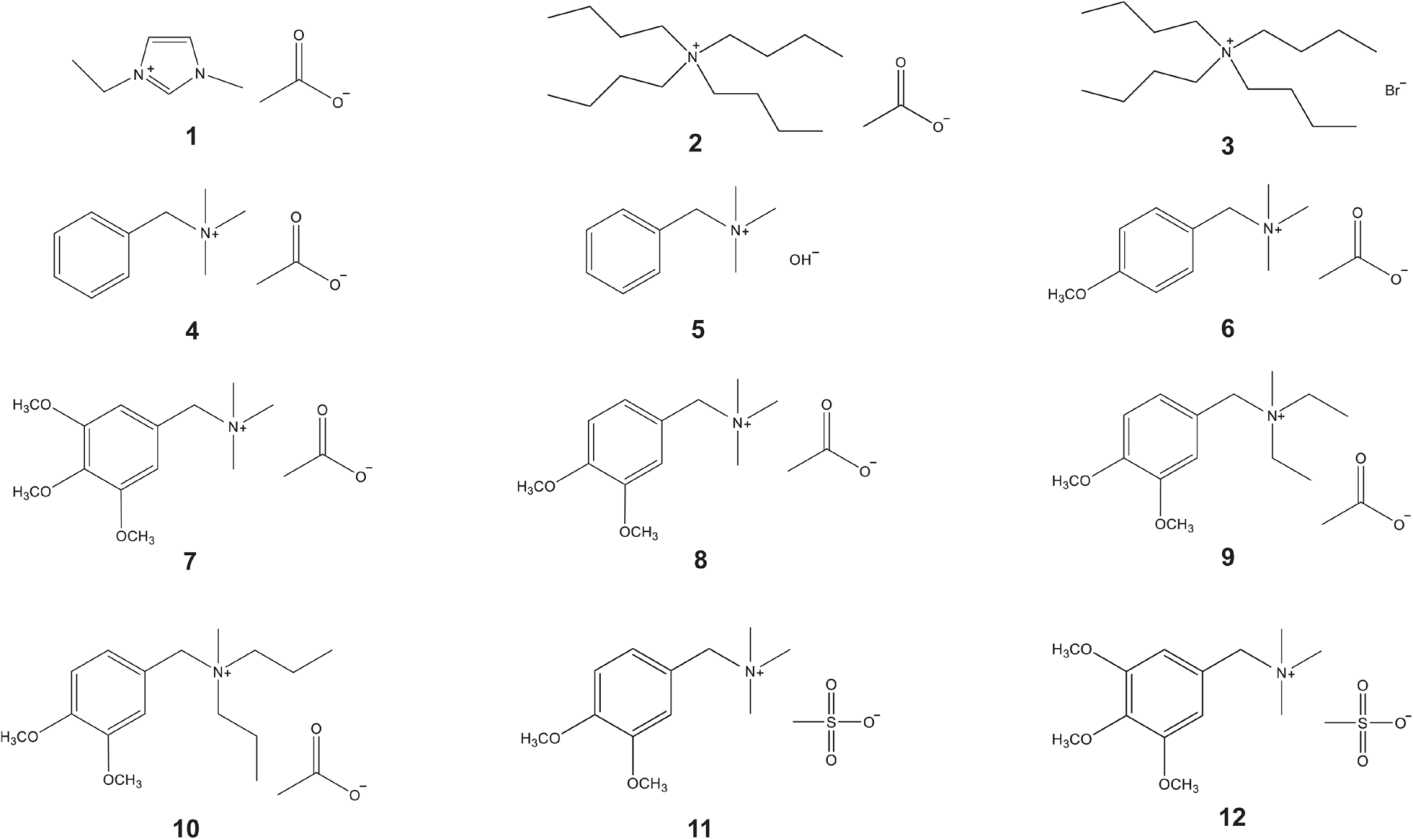
Structures of ionic liquids used in this study. Compounds **1**, **3**, **5** were used as controls to assess structure–activity relationships of compounds **2**, **4**, **6**–**12**

Of the lignin-derived ILs tested, those with elongated N-alkyl chains showed greater antibacterial activity, and those prepared from vanillin displayed the lowest toxicity against both *E. coli* and *D. magna* (Fig. 1).

## Results and discussion

### Synthesis of ionic liquids

Compound **1** was obtained from BASF, and compounds **3** and **5** were purchased from Sigma Aldrich. Compounds **2** and **4** were prepared from compounds **3** and **5**, respectively, using ion exchange. Compounds **6**–**8** were prepared using CBILS™ methyl carbonate chemistry as reported elsewhere [39]. Compounds **11** and **12** were synthesized from their corresponding methyl carbonates by ion exchange with methanesulfonic acid.

**Scheme 1.**
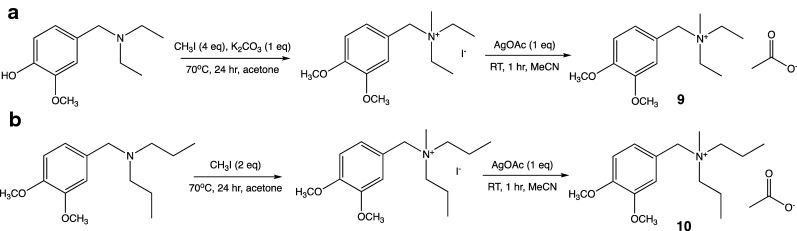
The synthesis of room-temperature ILs **9** and **10** from vanillin-derived benzylamines

**Fig. 2:**
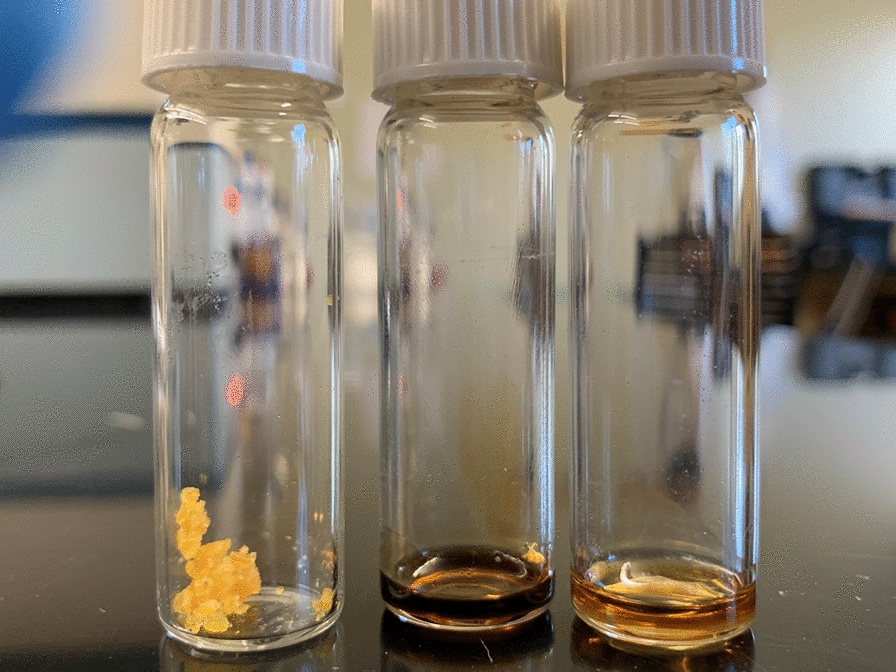
Compounds **8**–**10** (left to right) after vacuum drying at 80 °C and 0.3 Torr for 1 h

Synthesis of **9** was accomplished using the vanillin-derived benzylamine, 4-((diethylamino)methyl)-2-methoxyphenol [40]. Bismethylation with methyl iodide provided the isolable intermediate, *N*-(3,4-dimethoxybenzyl)-*N*-ethyl-*N*-methylethanaminium iodide. Lastly, **9** was achieved by ion exchange with silver acetate in 85% yield over 2 steps (Scheme 1a). Similarly, **10** was prepared from *N*-(3,4-dimethoxybenzyl)-*N*-propylpropan-1-amine using methyl iodide followed by silver acetate (77% yield over 2 steps) as shown in Scheme 1b. In contrast to **8**, which formed a solid at room temperature, **9** and **10** remained liquid after extensive vacuum drying (Fig. 2). This result corresponds to those observed for short chain alkyl and hydroxyalkyl ammonium ILs, whereby increasing *N*-alkyl chain length decreases melting point [46].

### Cellulose dissolution experiments

During biomass pretreatment with coordinating ILs, the crystalline nature of cellulose is often disrupted, providing downstream increases to saccharification rate and yield. Brønsted basic ILs provide hydrogen-bond-acceptor anions (e.g., carboxylate, dialkylphosphate, dialkylsulfonate) for cellulose hydroxyl groups, effectively swelling and/or solubilizing the cellulose polymer. Cellulose dissolution in quaternary ammonium ILs has been recently reviewed [47], showing a wide range of IL structures and cellulose solubilities, often involving co-solvents and catalysts (e.g., DMSO crown ethers). Neat tetraalkylammonium acetate ILs can dissolve approximately 2 wt% α-cellulose in 30 min at 100 °C [48]. Other systems, including *N,N*-allylmethylmorpholinium acetate, have shown up to 17 wt% dissolution of microcrystalline cellulose at 80 °C for 20 min [49]. Low temperature, short residence time cellulose solubility studies with ammonium ILs are limited, and to our knowledge, do not include benzyl ammonium cations.

**Fig. 3:**
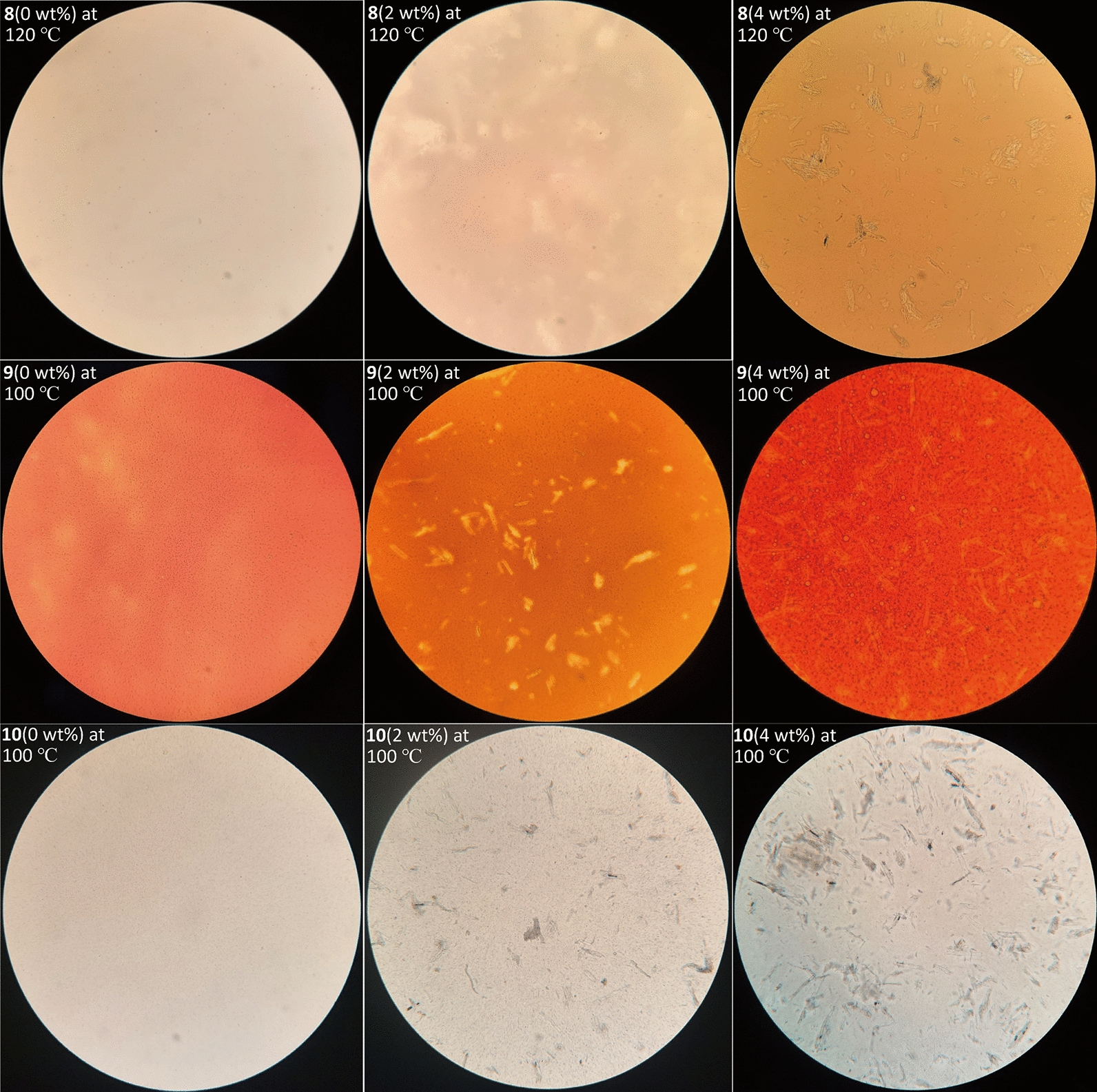
Light microscopy (10x) of lignin-derived ILs **8–10** with increasing cellulose concentrations

**Fig. 4:**
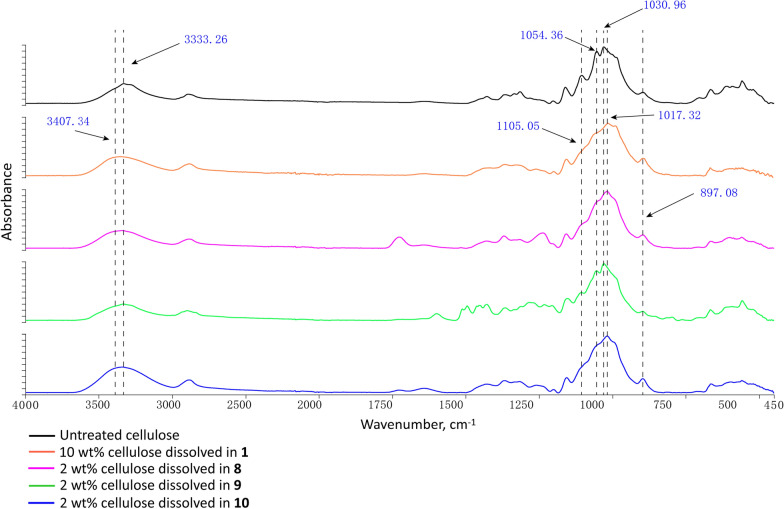
FTIR spectra of untreated microcrystalline cellulose, and cellulose precipitated from ILs **1**, **8**–**10**, after dissolution experiments

In this study, cellulose solubility in ILs **8**–**10** was determined gravimetrically, and light microscopy of ILs containing different concentrations of cellulose is provided in Fig. 3. For compound **8**, cellulose precipitation was observed at 4 wt% at 120 °C, which is in good agreement with previous studies of compound **8** at 100 °C [39]. Cellulose precipitation was readily observed in compounds **9** and **10** at 2 wt% at 100 °C. To further examine cellulose solubility, washed samples of cellulose precipitated from ILs **8**–**10** (2 wt%) were centrifuged and analyzed by FTIR. For spectral comparison, untreated microcrystalline cellulose, and cellulose dissolved in **1** (10 wt%) were also analyzed, and are shown in Fig. 4.

The FTIR spectra show subtle, yet distinct differences between untreated cellulose and IL-treated cellulose. The broad absorbance from 3600 to 3100 cm^−1^, can be assigned to the stretching vibration of the O–H covalent bond [50], and signal shift from 3333 cm^−1^ to 3407 cm^−1^ can be observed for cellulose recovered from ILs **1**, **8**, **10**. These results agree with work performed by Ciolacu et al. [51], who reported similar wavenumber increases corresponding to disruption of intra- and intermolecular H-bonds. The peak at 897 cm^−1^ represents C–O–C stretching at β-(1–4)-glycosidic linkages, an indicator for increased amorphous regions in the cellulose [50, 51]. As shown in Fig. 4, the peak at 897 cm^−1^ displayed stronger intensity in cellulose dissolved in **1**, **8**, **10**, and weaker intensity in untreated cellulose, and cellulose heated in **9**.

The peaks at 1105 cm^−1^ and 1054 cm^−1^ are, respectively, assigned to CH stretching vibrations and C–O–C skeletal vibration of cyclic polysaccharides [50, 52]. It can be seen from Fig. 4 that the intensities of these signals are also related to decreased cellulose crystallinity. The peak intensity at 1105 cm^−1^ is decreased in all dissolved cellulose samples. Additionally, decreased signal intensity at 1054 cm^−1^ was clearly observed in all IL dissolved cellulose, except for compound **9**. Lastly, the signal at 1031 cm^−1^, which is assigned to C–O–C stretching vibration for untreated cellulose [52], shifted to a lower wavenumber (1017 cm^−1^) in cellulose recovered from ILs **1**, **8** and **10**, yet this result is not observed in cellulose treated with **9**.

Based on the results from Figs. 3 and 4, it can be concluded that lignin-derived ILs **8** and **10** had the greatest ability to dissolve and decrease the crystallinity of cellulose, under the temperature and time conditions tested. Among the selected lignin-derived ILs, **9** demonstrated slightly less effective cellulose dissolution and decrystallization, which may have arisen from minor, yet persistent impurities as observed in the ^1^H NMR spectrum (Additional file 1: Figure S5).

### Antibiotic properties of selected ILs

**Fig. 5:**
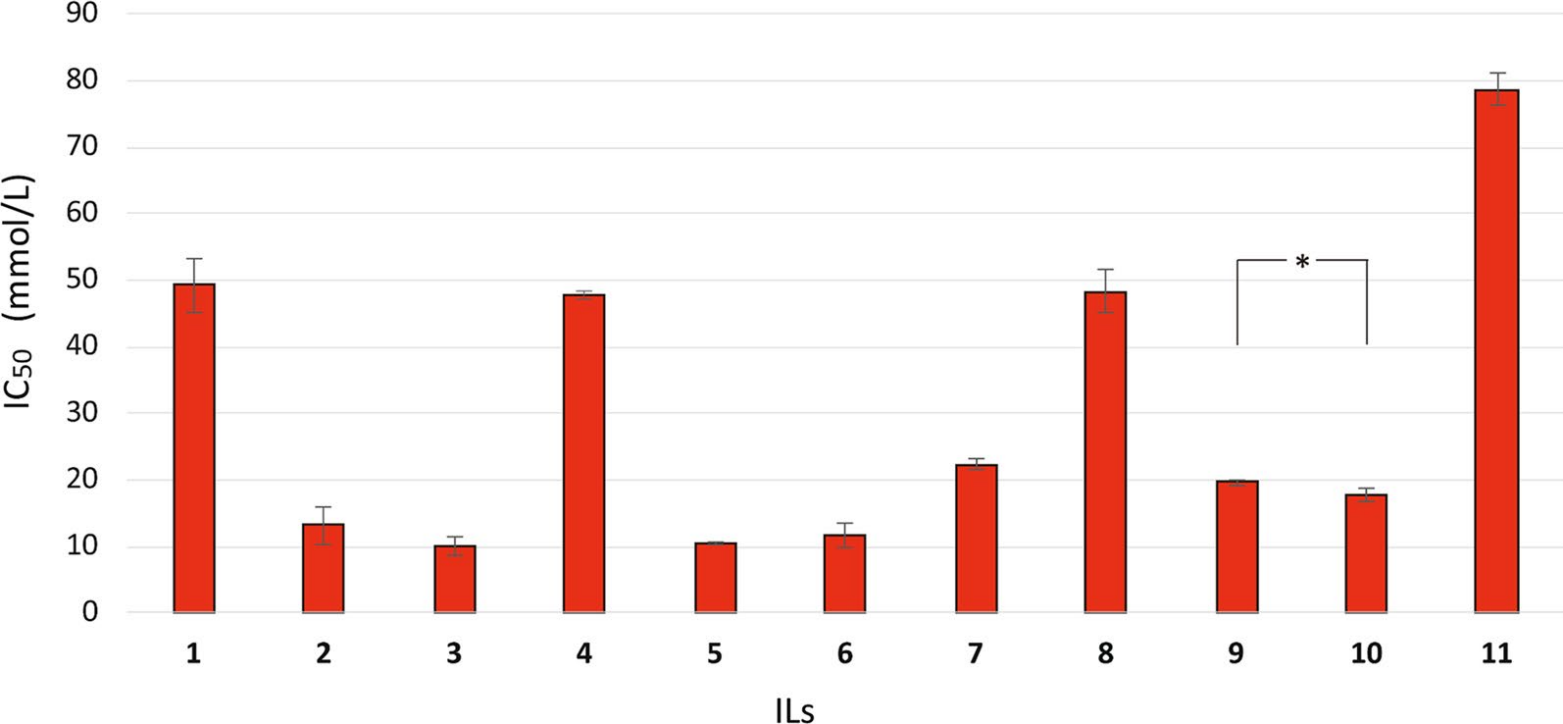
IC_50_ of ILs tested on *E. coli*. Asterisk: statistically significant difference (*p* = 0.002, *t* = 4.00) between **9** (*n* = 7) and **10** (*n* = 5)

The results of broth dilution assays with *E. coli* are shown in Fig. 5 as inhibitory concentration of IL required to decrease bacterial growth by 50% (IC_50_). The results indicate that IL toxicity is a function of both cation and anion structure. As expected, increasing the cation side chain lengths from *N,N*-dimethyl (**8**), *N,N*-diethyl (**9**) or *N,N*-dipropyl (**10**) increased the toxicity approximately 2.5-fold. Compound **10** (IC_50_ = 17.7 ± 0.9 mM) gave a slightly greater toxicity than **9** (IC_50_ = 19.6 ± 0.4 mM). This result can be explained by the increase of lipophilicity with the increase of the alkyl side chain length, which facilitates disruption of bacterial cell membranes. Though silver has well-known antibacterial properties, and is soluble in weakly coordinating ILs [53], washed ILs **9** and **10** did not show the characteristic signals of Ag^+^ using ESI-MS (Additional file 1: Figures S11-S13). Therefore, we conclude that the observed antibiotic activity is not due to the presence of residual silver in the ILs. Among the tetrabutylammonium ILs evaluated in this study, the acetate (**2**, IC_50_ = 13.2 ± 2.9 mM) showed slightly less toxicity when compared to the bromide (**3**, IC_50_ = 10.0 ± 1.3 mM, *p* = 0.055), and the toxicity of tetrabutylammonium ILs were generally greater than that of the benzylammonium ILs (**4**, **6**–**10**). Benzylammonium hydroxide (**5**) showed IC_50_ value of 10.4 ± 0.3 mM, and gave a pH measurement of 8.5 at this concentration in LB media. To determine if the antibacterial activity of **5** was due to pH effects, an additional broth dilution assay was performed using sodium hydroxide as a positive control. The IC_50_ value of NaOH was measured at 25.5 mM ± 0.4 mM, and the pH of the LB media at this concentration was measured at 9.4. Therefore, pH was not the major cause of antibacterial activity for compound **5**.

Interestingly, when holding the acetate anion, and the *N,N,N*-trimethyl side chains of the cation constant, the toxicity of benzyl trimethylammonium ILs (**4**, **6**–**8**) was modulated by the naturally occurring frequency and position of methoxy substitutions on the aromatic ring. A single methoxy substitution at the *para* position increased toxicity fourfold, as can be seen by comparing antibacterial values of compound **4** (IC_50_ = 47.8 ± 0.6 mM) to **6** (IC_50_ = 11.6 ± 1.7 mM). In compound **7**, derived from syringaldehyde, where methoxy groups are found in *para* and both *meta* positions, the IC_50_ value is 22.2 ± 0.8 mM. In compound **8**, derived from vanillin, a methoxy group occupies the *para* and a single *meta* position, and the IC_50_ is 48.2 ± 3.3 mM. The lignin-derived ILs are approximately 1000-fold less toxic as compared to longer chain alkyl benzylammonium salts (e.g., C_12_–C_16_), such as those widely used as disinfectants, which give bactericidal concentrations between 1 and 150 µM [54].

Anion type also affected the antibacterial activity of the lignin-derived ILs tested. Trimethyl benzylammonium acetate (**4**, IC_50_ = 47.8 ± 0.6 mM) was found to be fourfold less toxic than its corresponding hydroxide (**5**, IC_50_ = 10.4 ± 0.3 mM). Holding the *N,N,N*-trimethyl vanillin-derived cation constant, introduction of a methanesulfonate anion (**11**, IC_50_ = 78.7 ± 2.5 mM) reduced toxicity 1.6-fold as compared to the acetate anion (**8**, IC_50_ = 48.2 ± 3.3 mM). Therefore, it can be concluded that the toxicity of anions showed a trend of methanesulfonate < acetate < hydroxide for *N,N,N*-trimethyl benzylammonium ILs. Similar results were obtained by Wood et al. [33], whereby ionic liquids prepared from alkylammonium cations and sulfate and sulfonate anions were found to be non-toxic in disk-diffusion assays using *E. coli*.

Analogous structure–activity relationships were observed from lignin-derived cations in acute toxicity assays with *D. magna*. The least antibacterial anion, methanesulfonate, was selected for evaluation, and paired with cations derived from G- and S-type lignin (**11** and **12**, respectively). Similar to the antibacterial trend, syringaldehyde-derived IL **12** (IC_50_ = 0.123 ± 0.012 mM) was 2.6-fold more toxic to *D. magna* than vanillin-derived IL **11** (0.319 ± 0.066 mM). It should be noted that the IC_50_ of **11** (0.319 mM) against *D. magna* is approximately 250-fold lower than its IC_50_ against *E. coli* (78.7 mM) indicating that *D. magna* is far more sensitive to *N,N,N*-trimethyl benzylammonium ILs. These data are in good agreement to reported values for *D. magna* (IC_50_ 0.1–0.5 µM), using longer chain (e.g., C_12_–C_16_) alkyl benzylammonium salts. [54]

## Conclusion

The antibacterial effects of quaternary ammonium salts has been known for over a century, but increased production and usage during the COVID-19 pandemic [54] coupled to resistance in Gram-negative bacteria such as *P. aeruginosa* and *E. coli* [55] warrants investigation of bio-based alternatives. New synthetic approaches to lignin-derived quaternary ammonium compounds provided room-temperature ILs **9** and **10** that demonstrated rapid cellulose dissolution capacity at 100 °C. FTIR analysis confirmed that cellulose dissolution also reduced cellulose crystallinity. Broth dilution assays with ILs and *E. coli* suggested that antibacterial activity was due to both the structure of cations and type of anions. Anion toxicity followed the trend of methanesulfonate < acetate < hydroxide. As compared to ILs derived from syringaldehyde and 4-methoxybenzaldehyde, asymmetric methoxy substitution on the benzyl ring of the cation may have reduced the toxicity of vanillin-derived ILs. The antibacterial mechanism of action for compounds **9** and **10** is likely due to IL disruption of the lipopolysaccharide cell membrane, as evidenced by the trend of increasing toxicity with increasing cation *N*-alkyl chain length. The reduced toxicity of ILs derived from vanillin (**8**, **11**) against both *E. coli* and *D. magna* suggests that ILs derived from softwood lignin (G-type) could be favorable pretreatment solvents for biofuels produced with integrated fermentations, and/or bioproducts. These results warrant additional evaluation of room-temperature, lignin-derived ILs for biomass pretreatment, including lignin removal, effect on enzymatic saccharification and biodegradation.

## Methods

NMR spectra were acquired by a Bruker Advance III HD spectrometer at the frequency of 400 MHz for ^1^H (100 MHz for ^13^C). High-resolution mass spectrometry (HR-MS) was performed on an Agilent accurate-mass 6520B Q-TOF mass spectrometer. A Perkin Elmer Spectrum Two FTIR spectrometer equipped with a universal diamond ATR unit was used for the analysis of dissolved cellulose. Spectra of samples were recorded between range of 450 cm^−1^ and 4500 cm^−1^, at a resolution of 4 cm^−1^. The spectra shown in Fig. 4 represent the accumulation of 4 scans/sample with baseline correction applied. All purchased chemicals were used without purification.

### Procedure for the synthesis of ILs 2, 4, 9 and 10.

Tetrabutylammonium acetate (**2**) was prepared by dissolving tetrabutylammonium bromide (**3**) (322 mg, 1 mmol, 1 eq) of in 20 mL of acetonitrile. Silver acetate (167 mg, 1 mmol, 1 eq) was then added to the mixture. The reaction was allowed to stir at RT for 1 h. After the reaction, the solution was centrifuged at 4000 × G for 10 min, and the supernatant was collected and dried under vacuum (0.3 Torr, 1 h) at 50 °C. The product was obtained as a light yellow oil in 89% yield. ^1^H NMR (acetone -*d*_6_): 0.97 (12H, t), 1.41 (8H, m), 1.68 (3H, s), 1.78 (8H, m), 3.48 (8H, t). ^13^C NMR: 13.93, 20.34, 24.50, 25.81, 59.14, 59.16, 174.47. HR-MS: [C_16_H_36_N]^+^, found 242.2845, calcd. 242.2842 (1.2 ppm). The ^1^H and ^13^C NMR of compound **2** are shown in Additional file 1: Figures S1, S2.

Benzyltrimethylammonium acetate (**4)** was prepared by mixing 418 mg (1 mmol, 1 eq) of benzyltrimethylammonium hydroxide (**5**, 40 wt% aqueous solution) with 60 mg glacial acetic acid (1 mmol, 1 eq). The reaction was allowed to stir at RT for 1 h. After the reaction, the solution was dried under vacuum (0.3 Torr, 1 h) at 50 °C. The product was obtained as a clear liquid in quantitative yield. ^1^H NMR (D_2_O): 1.86 (3H, s), 3.06 (9H, s), 4.44 (2H, s), 7.52 (5H, m). ^13^C NMR: 23.42, 52.36, 52.40, 52.44, 69.63, 127.43, 129.25, 130.90, 132.86, 181.29. HR-MS: [C_10_H_16_N]^+^, found 150.1278, calcd. 150.1277 (0.7 ppm). The ^1^H and ^13^C NMR of compound **4** are shown in Additional file 1: Figures S3, S4.

*N*-(3,4-Dimethoxybenzyl)-*N*-ethyl-*N*-methylethanaminium acetate (**9**) was prepared by dissolving 209 mg (1 mmol, 1 eq) of 4-((diethylamino)methyl)-2-methoxyphenol in 2 mL of acetone. Dry K_2_CO_3_ (138 mg, 1 mmol, 1 eq) was added, followed by methyl iodide (568 mg, 4 mmol, 4 eq). The reaction was kept at 70 °C for 24 h. After the reaction, the solution was filtered and then dried under vacuum (0.3 Torr, 1 h) at 50 °C. The product was then re-dissolved in 20 mL of acetonitrile and mixed with silver acetate (167 mg, 1 mmol, 1 eq). After 1 h of reaction at RT, the solution was centrifuged at 4000 × G for 10 min, and the supernatant was collected and dried under vacuum (0.3 Torr, 1 h) at 50 °C. To ensure the complete removal of the insoluble silver salts, the dried product was washed with 10 mL of methanol and then filtered through 0.25 mm syringe filter. The filtrate was then dried under vacuum (0.3 Torr, 1 h) at 50 °C. The final product was obtained as a dark brown oil in 85% overall yield. ^1^H NMR (DMSO-*d*_6_ with 1 µL acetone as calibration standard): 1.19 (6H, t), 1.73 (3H, s), 2.96 (6H, s), 3.29 (4H, q), 3.71 (3H, m), 4.91 (2H, m), 6.86 (3H, m). ^13^C NMR: 7.42, 24.29, 48.52, 55.17, 56.66, 57.54, 73.71, 112.17, 115.04, 120.40, 127.22, 147,41, 147.53, 173.10. HR-MS: [C_14_H_24_NO_2_]^+^, found 238.1803, calcd. 238.1802 (0.4 ppm). The ^1^H and ^13^C NMR of compound **9** are shown in Additional file 1: Figures S5, S6.

*N*-(3,4-Dimethoxybenzyl)-*N*-propylpropan-1-amine was prepared by dissolving 3,4-dimethoxybenzaldehyde (10.0 g, 60 mmol, 1 eq) into 200 mL dry acetonitrile. While stirring, dipropylamine (7.92 g, 78 mmol, 1.3 eq) was added followed by sodium triacetoxyborohydride (17.85 g, 84.2 mmol, 1.4 eq). The reaction was allowed to stir at RT overnight and worked up as reported elsewhere [38]. After vacuum drying (0.3 Torr, 1 h) at 50 °C, the product (14.1 g, 55.8 mmol) was obtained as a pale yellow oil in 93% yield. ^1^H NMR (CDCl_3_): 0.86 (6H, t), 1.49 (4H, m), 2.40 (4H, t), 3.53 (2H, s), 3.86 (3H, s), 3.88 (3H, s), 6.79 (2H, m), 6.99 (1H, s). ^13^C NMR: 11.99, 19.97, 55.61, 55.94, 55.98, 58.40, 110.76, 112.09, 120.92, 148.01, 148.95. HR-MS: [C_15_H_26_NO_2_]^+^, found 252.1955, calcd. for 252.1958 (1.2 ppm). The ^1^H and ^13^C NMR of *N*-(3,4-dimethoxybenzyl)-*N*-propylpropan-1-amine are shown in Additional file 1: Figures S7, S8.

*N*-(3,4-Dimethoxybenzyl)-*N*-methyl-*N*-propylpropan-1-ammonium acetate (**10**) was prepared by dissolving *N*-(3,4-dimethoxybenzyl)-*N*-propylpropan-1-amine (325 mg, 1 mmol) in 2 mL of acetone. Methyl iodide (284 mg, 2 mmol, 2 eq) was then added to the mixture. The reaction was kept at 70 °C for 24 h. After the reaction, the solution was filtered and dried under vacuum (0.3 Torr, 1 h) at 50 °C, and the product was then re-dissolved in 20 mL of acetonitrile and mixed with silver acetate (167 mg, 1 mmol, 1 eq). After 1 h of reaction at RT, the solution was centrifuged at 4000 × G for 10 min, and the supernatant was collected and dried under vacuum (0.3 Torr, 1 h) at 50 °C. To ensure the complete removal of the insoluble silver salts, the dried product was washed with 10 mL of methanol and then filtered through 0.25 mm syringe filter. The filtrate was then dried under vacuum (0.3 Torr, 1 h) at 50 °C. The final product was obtained as a dark brown oil in 77% overall yield. ^1^H NMR: 0.89 (6H, t), 1.60 (3H, s), 1.75 (4H, m), 2.92 (3H, s), 3.14 (4H, m), 3.78 (6H, s), 4.52(2H, s), 7.09 (3H, m). ^13^C NMR (DMSO-*d*_6_): 10.58, 15.28, 25.58, 46.82, 48.43, 55.51, 55.67, 61.31, 64.44, 111.54, 116.34, 120.18, 125.90, 148.54, 150.12, 173.18. HR-MS: [C_16_H_28_NO_2_]^+^, found 266.2118, calcd. 266.2115 (1.1 ppm). The ^1^H and ^13^C NMR of compound **10** are shown in Additional file 1: Figures S9, S10.

### Cellulose dissolution

Avicel PH 101 microcrystalline cellulose obtained from Sigma Aldrich was used in the dissolution experiments. To test cellulose solubility, 500 mg of selected ILs were collected in a 7 mL transparent glass tube and heated in a sand bath at 100 °C for 1 h (compound **8** was heated to 120 °C for 1 h for due to its higher melting point). Cellulose (10 mg, 2 wt%) was added slowly into the tube, and the solution was stirred with a spatula. After 20 min of incubation, a drop of the solution was taken and observed under a light microscope (10 × magnification). The procedure was repeated with 20 mg (4%) of total cellulose addition.

### FTIR spectroscopy of dissolved cellulose

The IL solutions with 2 wt% cellulose concentration were used for FTIR analysis. Methanol (5 mL) was added into the tube to dissolve the IL and precipitate the dissolved cellulose. The solution was then transferred to a 50 mL centrifuge tube and centrifuged at 4000 × G for 10 min. After removal of the supernatant, the cellulose at bottom was dried in an oven at 70 °C for 1 h before FTIR analysis.

### Antibiotic assays

*E. coli* strain 1A1 [43] was obtained from the microbial strain repository at the Joint Bioenergy Institute at Lawrence Berkeley National Laboratory. A 10 mL culture in Luria Broth (LB) media was grown for 18 h at 37 °C with shaking at 200 rpm. The culture was then diluted into LB media to an absorbance of 0.05 at 600 nm, and added into the wells of a 96-well plate (first row = 190 µL, all other wells = 100 µL/well). Approximately 700 mg of IL was dissolved into 1 mL of methanol, and 10 µL of each IL solution was added to first row wells of the plate. A twofold serial dilution was performed down the plate by mixing and transferring 100 µL using a multi-channel pipette. To maintain isochoric conditions, 100 µL was discarded from bottommost wells. The plate was then incubated at 37 °C with shaking at 150 rpm. After 24 h incubation, *E. coli* growth was quantified at 600 nm using an Epoch Microplate Spectrophotometer (Gen5 software). Cells grown with 10 µL of methanol were used as negative controls, and resulted in zero growth inhibition. All experiments were repeated 3–7 times and error bars in Fig. 5 represent standard deviation. Statistical F-test was performed to determine the appropriate t-test (i.e., equal or unequal variance) to analyze significant difference between IC_50_ data.

### *Daphnia magna* cultivation

Procedures for cultivating and performing acute toxicity assays with *D. magna* generally followed the US EPA protocol [56]. *Daphnia magna* was cultured from stocks supplied by Aquatic Bio Systems Inc. (Fort Collins, CO). The organisms were cultured in 900-mL jars filled with moderately hard water from the recipe: 0.473 g CaSO_4_, 0.959 g NaHCO_3_, 1.223 g MgSO_4_·7H_2_O, and 0.039 g KCl per 10 L of deionized water. 2 mL of algae (*Selenastrum capriconutum*) containing 3.0 × 10^7^ cells/mL were fed to *D. magna* on both Tuesdays and Thursdays with water changes as well as feedings on Saturdays. All glass jars were placed in a Thermo Scientific Precision incubator (Fischer Scientific, Hampton, NH) at a holding temperature of 20 °C and on a 12 h:12 h light:dark cycle. This procedure was conducted approximately a month prior to the beginning of the project. To prepare *D. magna* for use in acute experiments, three hundred adult daphnids were separated into five 1-L beakers with 60 daphnids each. These daphnids were used a week later in the experiment. All daphnid cultures followed the same protocol for feeding and water changes as mentioned previously.

### Acute toxicity study of ILs on *D. magna*

A 48-h acute toxicity study was carried out to examine the toxic effects of **11** and **12** on *D. magna*, Glass jars were filled with moderately hard water and spiked with the appropriate concentration of IL from stock solution to a total volume of 50 mL. For each IL, 5 different concentrations were studied. Control jars were set up with only 50 mL of moderately hard water. Four replicates were used for each concentration and each replicate had four daphnids. The daphnids were placed into glass jars for each concentration of ILs. All glass jars were placed in a Thermo Scientific Precision incubator (Fischer Scientific, Hampton, NH) at a holding temperature of 20 °C and on a 12 h:12 h light:dark cycle. Mortality in each jar was recorded every 24 h.

## Supplementary Information

**Additional file 1.** **Fig. S1**. ^1^H NMR spectrum of compound **2** dissolved in acetone-d_6_ containing TMS (400 MHz). **Fig. S2** ^13^C NMR spectrum of compound **2** dissolved in acetone-d_6_ containing TMS (400 MHz). **Fig. S3** ^1^H NMR spectrum of compound **4** dissolved in D_2_O (400 MHz). **Fig. S4** ^13^C NMR spectrum of compound **4** dissolved in D_2_O (400 MHz). **Fig. S5** ^1^H NMR spectrum of compound **9** dissolved in DMSO-d_6_ (400 MHz). **Fig. S6** ^13^C NMR spectrum of compound **9** dissolved in DMSO-d_6_ (400 MHz). **Fig. S7** ^1^H NMR spectrum of N-(3,4-dimethoxybenzyl)-N-propylpropan-1-amine dissolved in CDCl_3_ (400 MHz). **Fig. S8** ^13^C NMR spectrum of N-(3,4-dimethoxybenzyl)-N-propylpropan-1-amine dissolved in CDCl_3_ (400 MHz). **Fig. S9** ^1^H NMR spectrum of compound **10** dissolved in DMSO-d_6_ (400 MHz). **Fig. S10** ^1^3C NMR spectrum of compound **10** dissolved in DMSO-d_6_ (400 MHz). **Fig. S11** Low molecular weight regions of ESI-MS spectra for compound **9** in positive ion mode showing the absence of residual Ag^+^ ion. **Fig. S12** Low molecular weight regions of ESI-MS spectra for compound **10** in positive ion mode showing the absence of residual Ag^+^ ion. **Fig. S13** ESI-MS spectrum of control spectrum showing Ag^+^ signals at 106.9047 (100%) and 108.9043 (92.9%).

## Data Availability

Data and materials used in this study are available upon request.
