# Supplementary material for: Synthesis, antibiotic structure–activity relationships, and cellulose dissolution studies of new room-temperature ionic liquids derived from lignin
Source: Biotechnol Biofuels. 2021 Feb 23;14:47. doi: 10.1186/s13068-021-01898-x (PMC7900799; doi:10.1186/s13068-021-01898-x)
Supplement: Supplementary file 1 — Additional file 1. Fig. S1. 1H NMR spectrum of compound 2 dissolved in acetone-d6 containing TMS (400 MHz). Fig. S2 13C NMR spectrum of compound 2 dissolved in acetone-d6 containing TMS (400 MHz). Fig. S3 1H NMR spectrum of compound 4 dissolved in D2O (400 MHz). Fig. S4 13C NMR spectrum of compound 4 dissolved in D2O (400 MHz). Fig. S5 1H NMR spectrum of compound 9 dissolved in DMSO-d6 (400 MHz). Fig. S6 13C NMR spectrum of compound 9 dissolved in DMSO-d6 (400 MHz). Fig. S7 1H NMR spectrum of N-(3,4-dimethoxybenzyl)-N-propylpropan-1-amine dissolved in CDCl3 (400 MHz). Fig. S8 13C NMR spectrum of N-(3,4-dimethoxybenzyl)-N-propylpropan-1-amine dissolved in CDCl3 (400 MHz). Fig. S9 1H NMR spectrum of compound 10 dissolved in DMSO-d6 (400 MHz). Fig. S10 13C NMR spectrum of compound 10 dissolved in DMSO-d6 (400 MHz). Fig. S11 Low molecular weight regions of ESI-MS spectra for compound 9 in positive ion mode showing the absence of residual Ag+ ion. Fig. S12 Low molecular weight regions of ESI-MS spectra for compound 10 in positive ion mode showing the absence of residual Ag+ ion. Fig. S13 ESI-MS spectrum of control spectrum showing Ag+ signals at 106.9047 (100%) and 108.9043 (92.9%). [file 13068_2021_1898_MOESM1_ESM.docx]

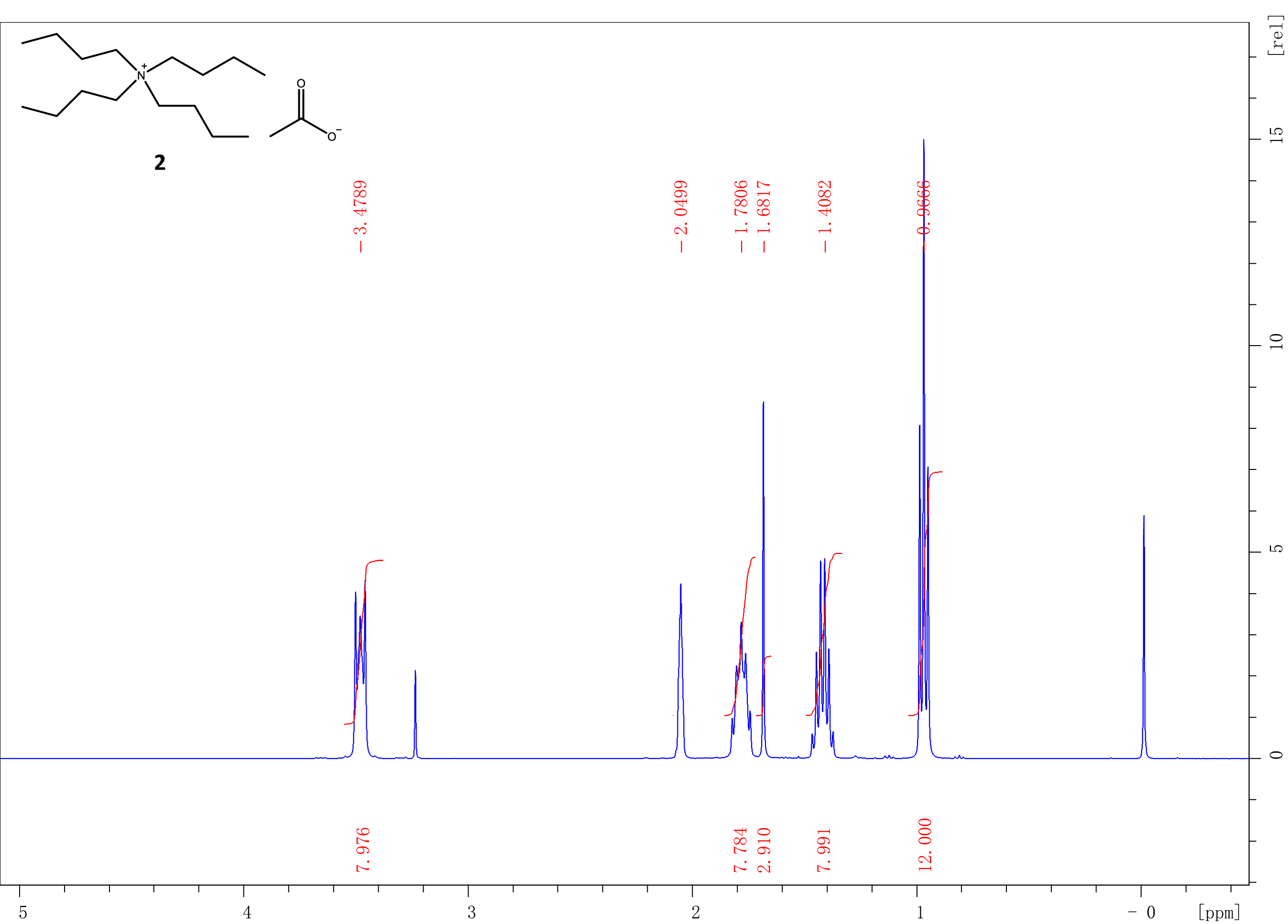


**Figure S1.** ^1^H NMR spectrum of compound **2** dissolved in acetone-d_6_ containing TMS (400 MHz).


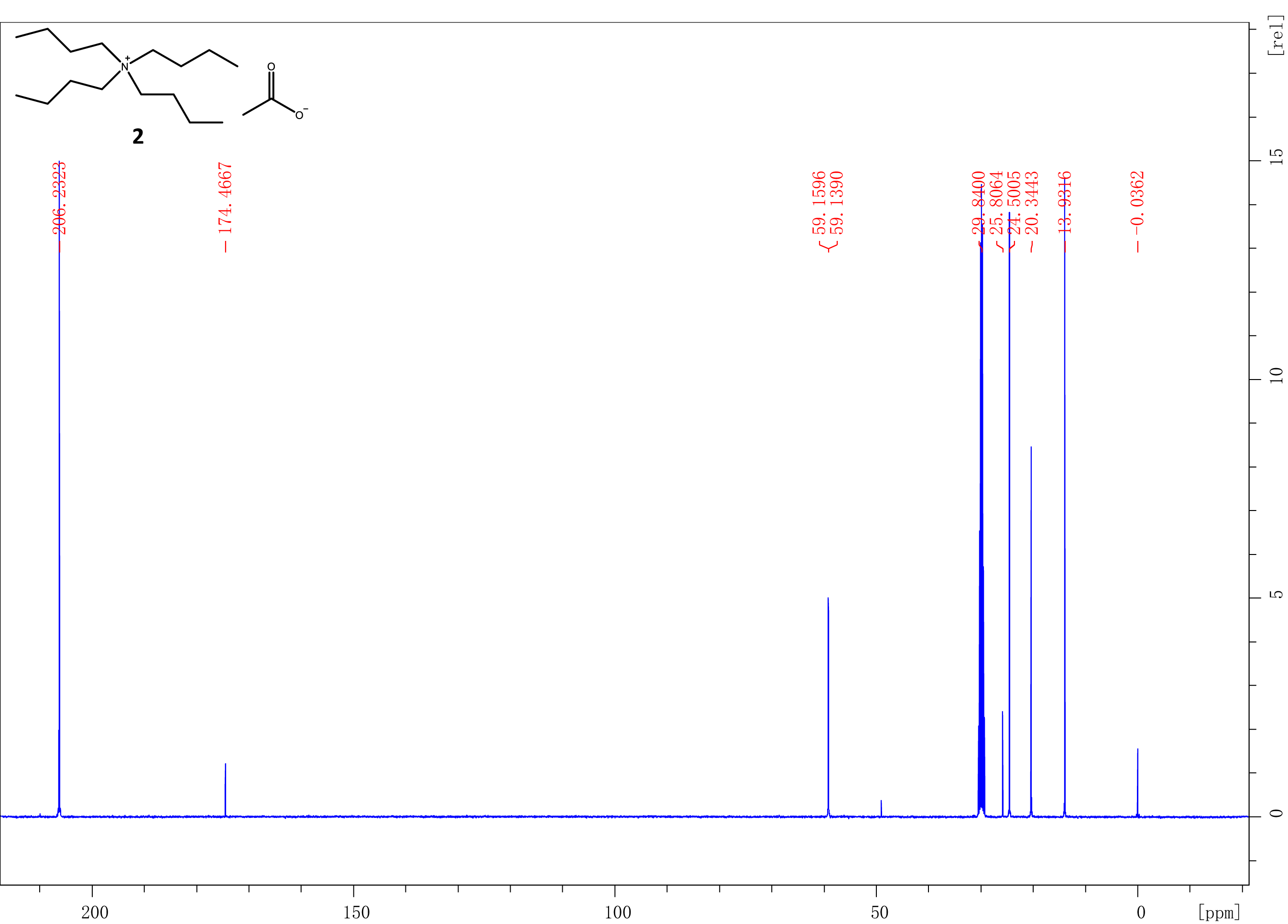


**Figure S2.** ^13^C NMR spectrum of compound **2** dissolved in acetone-d_6_ containing TMS (400 MHz).


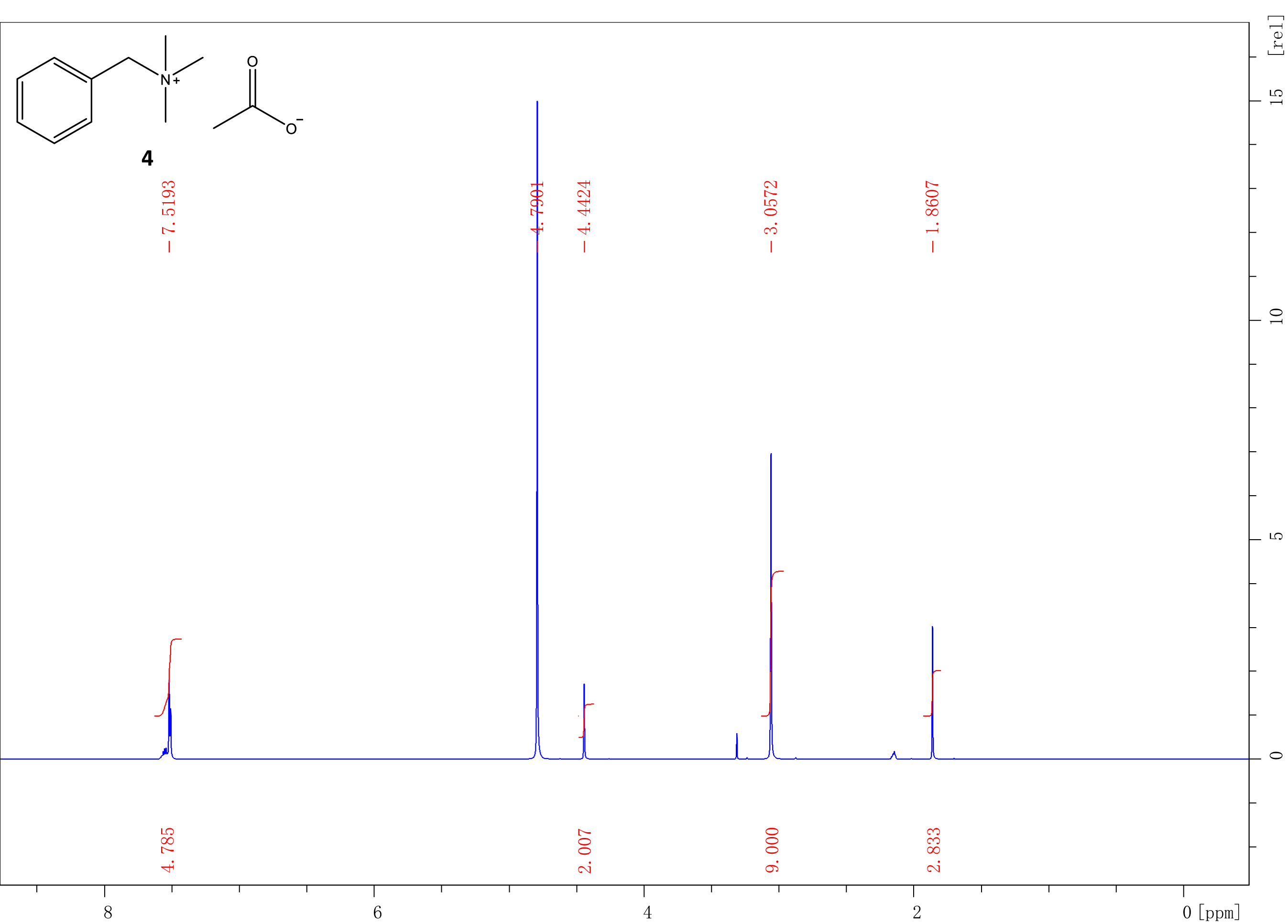


**Figure S3.** ^1^H NMR spectrum of compound **4** dissolved in D_2_O (400 MHz).


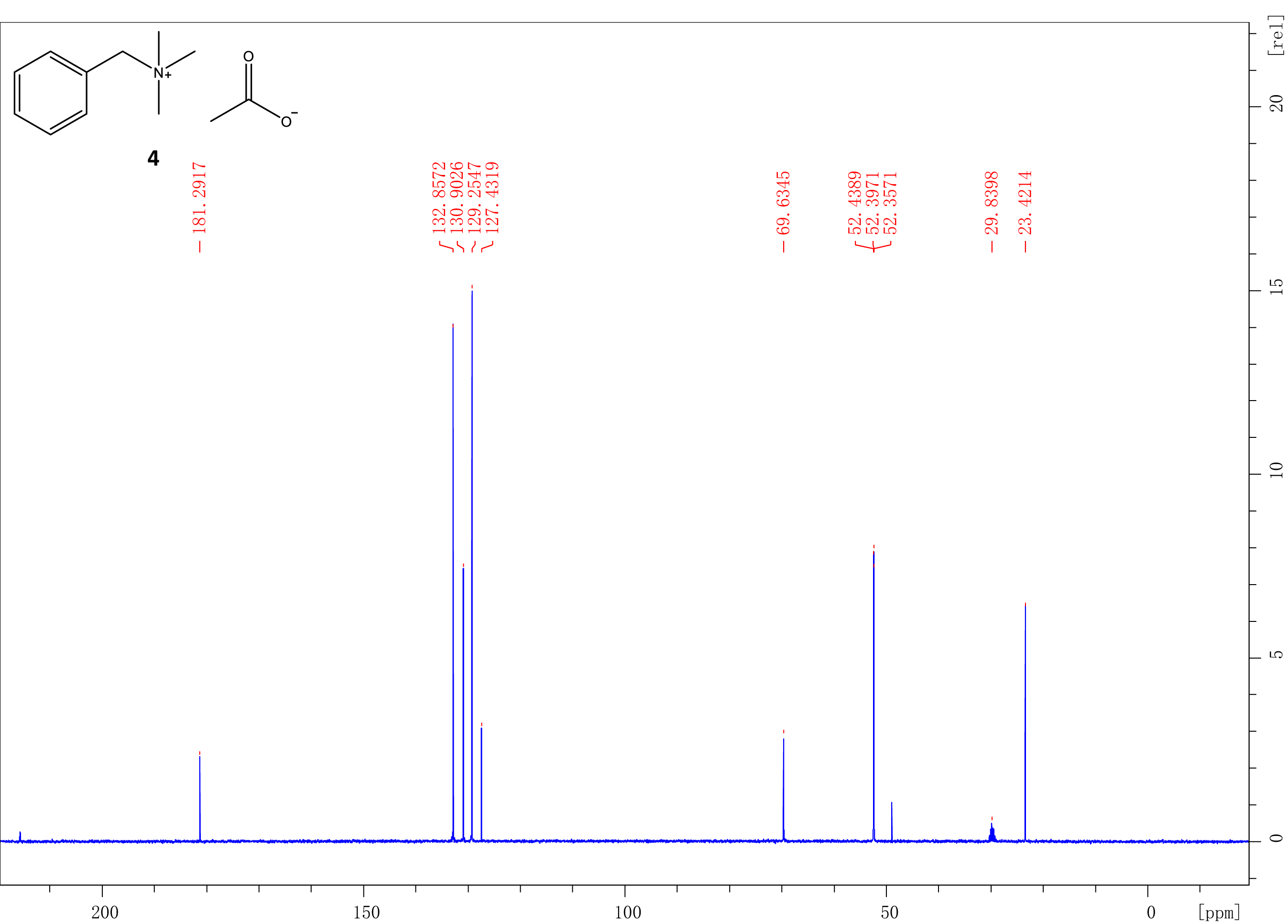


**Figure S4.** ^13^C NMR spectrum of compound **4** dissolved in D_2_O (400 MHz).


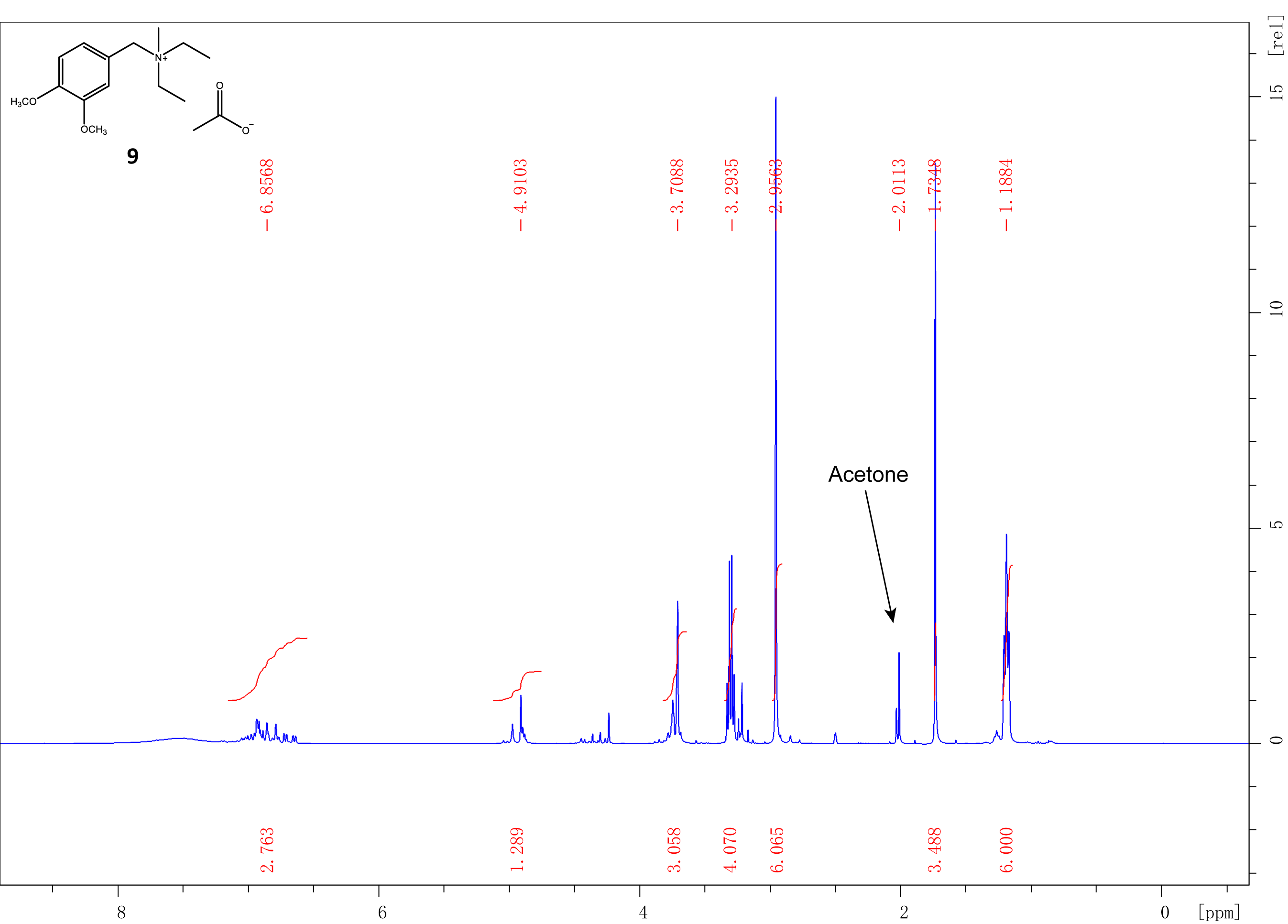


**Figure S5.** ^1^H NMR spectrum of compound **9** dissolved in DMSO-d_6_ (400 MHz).


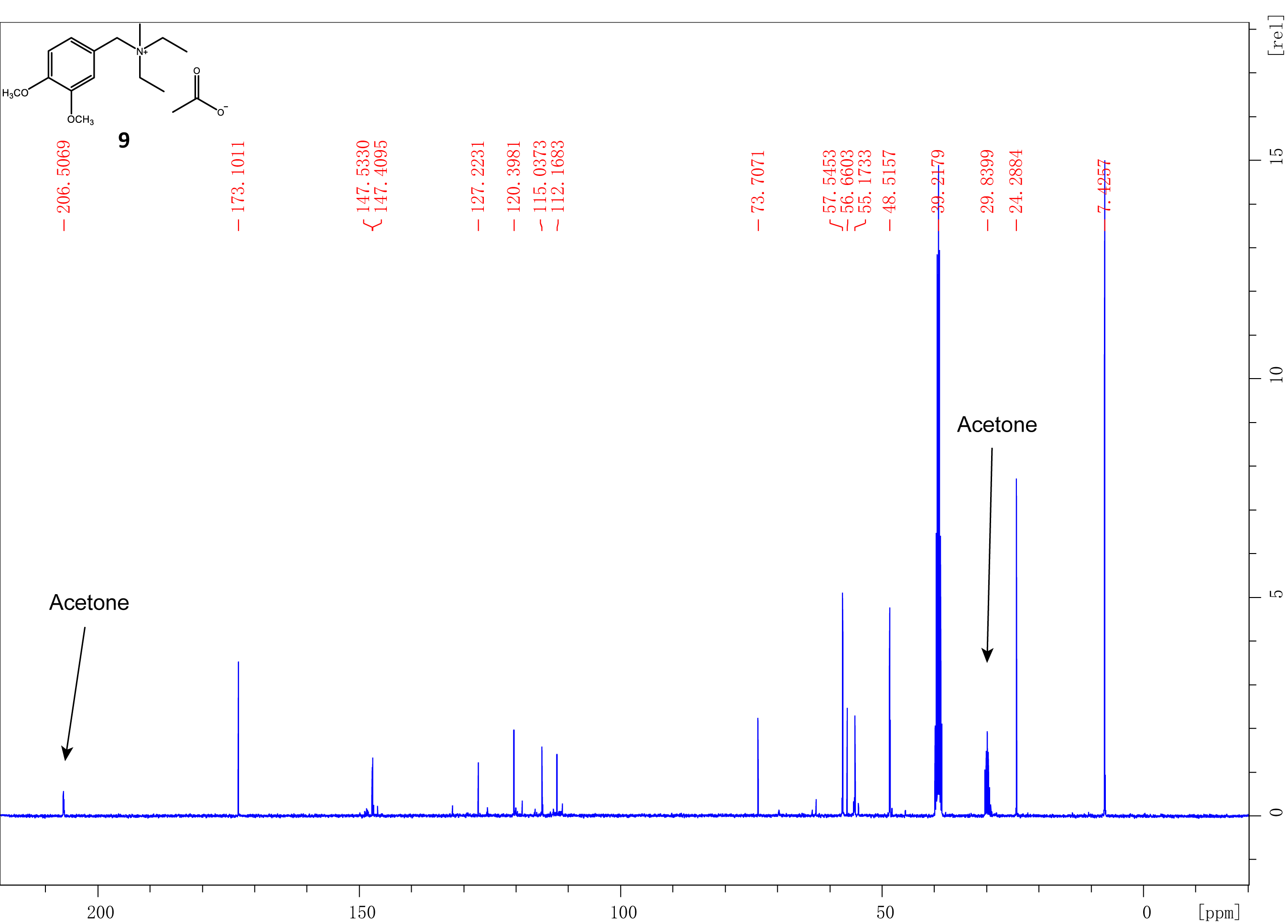


**Figure S6.** ^13^C NMR spectrum of compound **9** dissolved in DMSO-d_6_ (400 MHz).


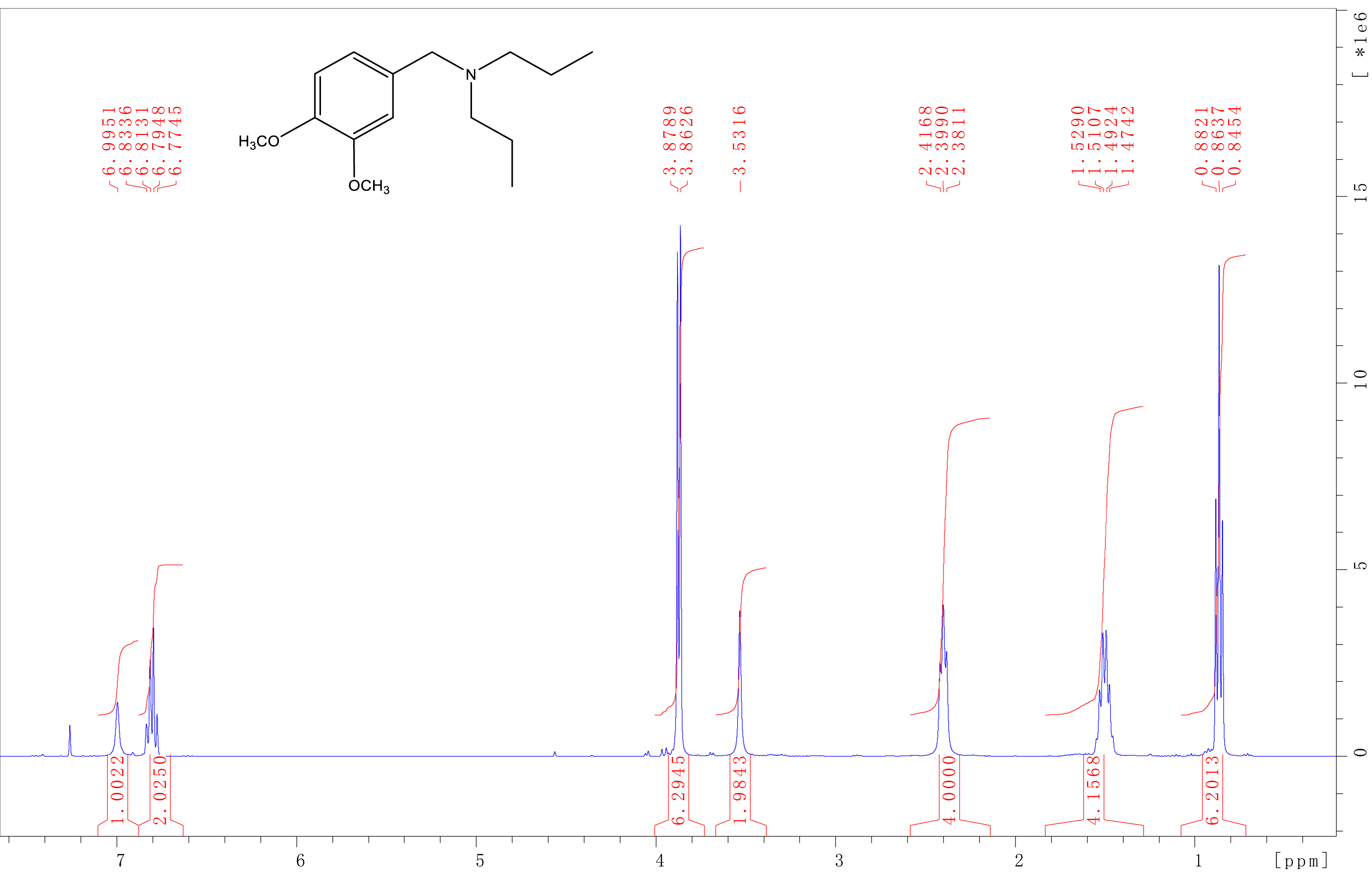


**Figure S7.** ^1^H NMR spectrum of N-(3,4-dimethoxybenzyl)-N-propylpropan-1-amine dissolved in CDCl_3_ (400 MHz).


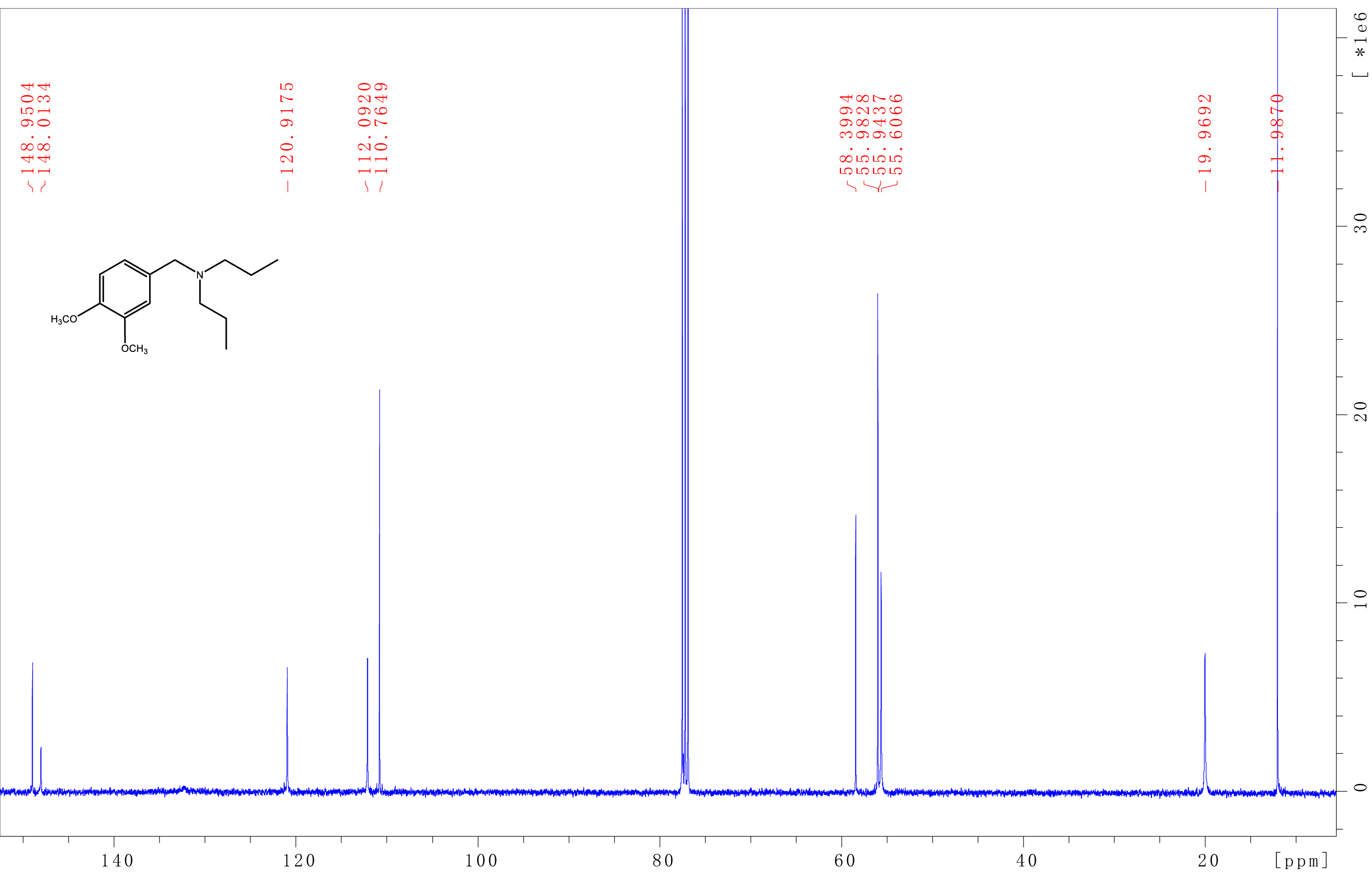
**Figure S8.** ^13^C NMR spectrum of N-(3,4-dimethoxybenzyl)-N-propylpropan-1-amine dissolved in CDCl_3_ (400 MHz).

**
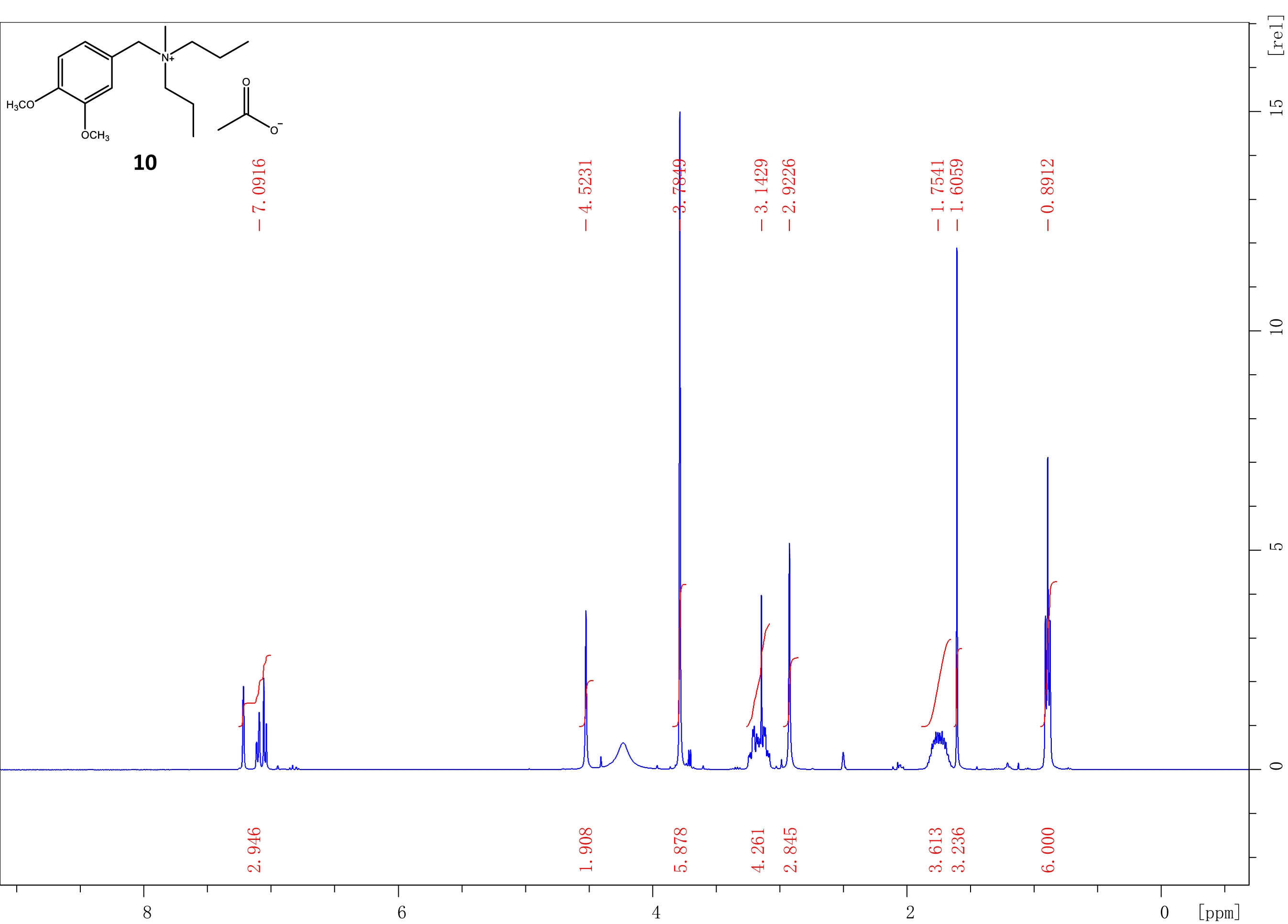
**

**Figure S9.** ^1^H NMR spectrum of compound **10** dissolved in DMSO-d_6_ (400 MHz).


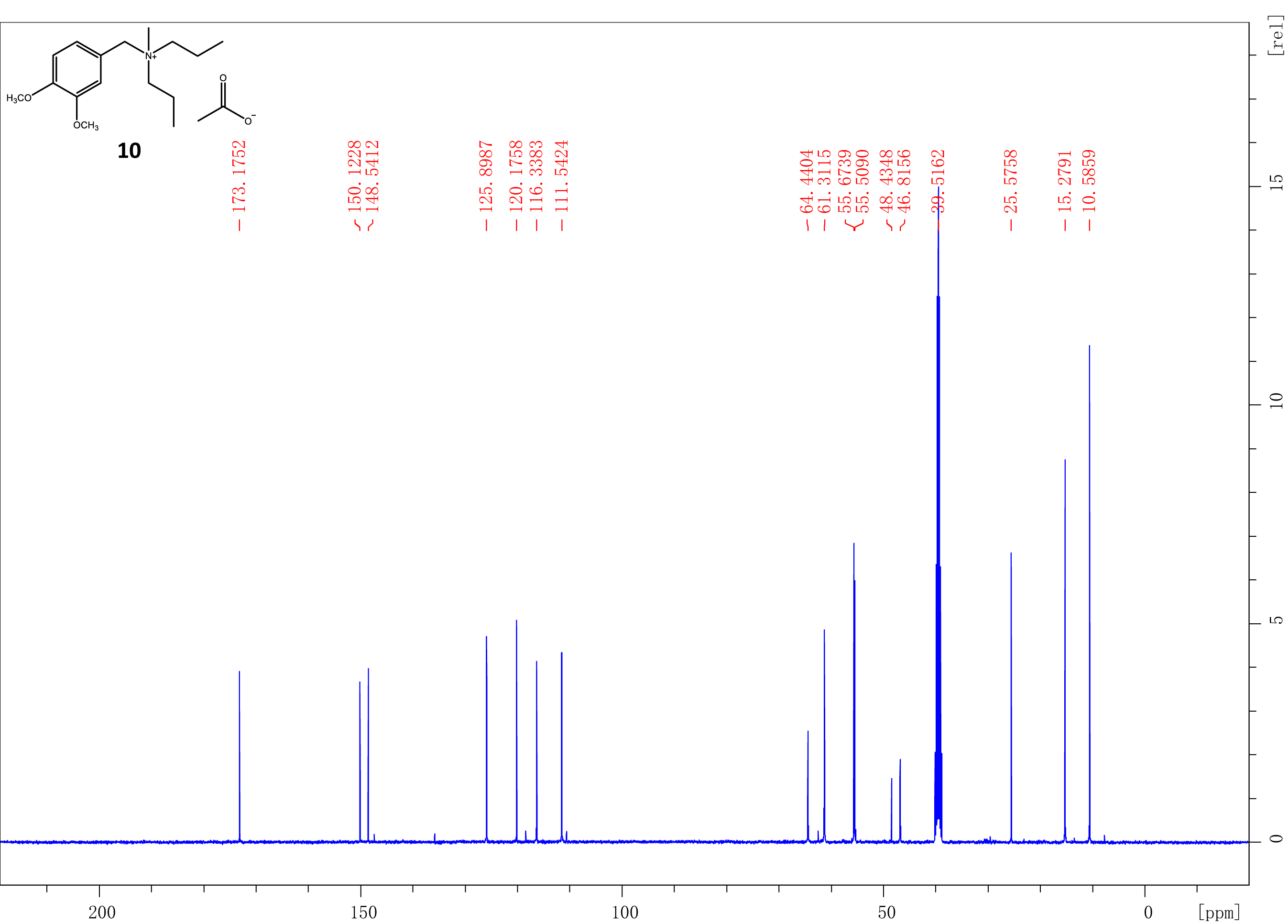


**Figure S10.** ^13^C NMR spectrum of compound **10** dissolved in DMSO-d_6_ (400 MHz)


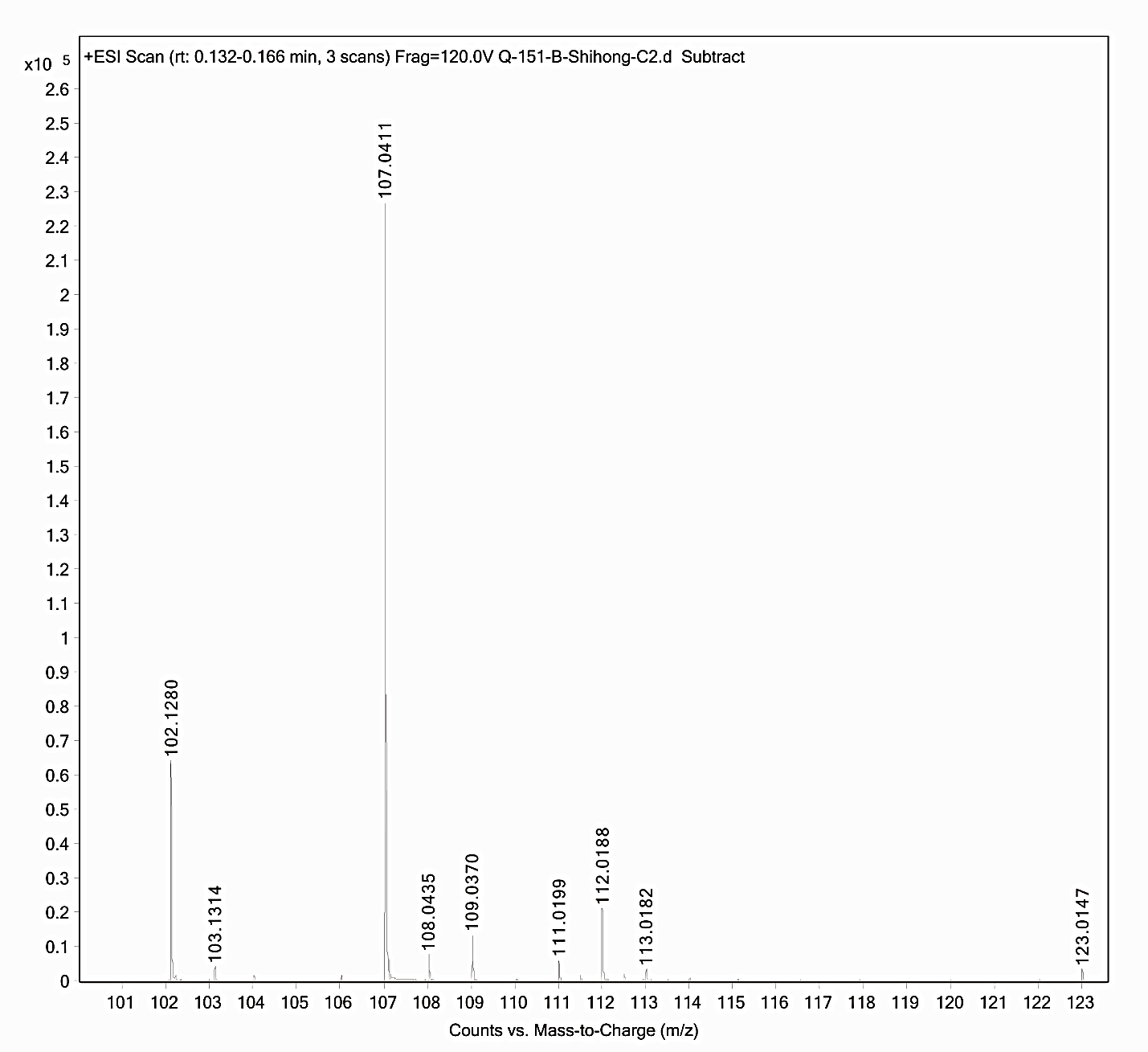


**Figure S11**. Low molecular weight regions of ESI-MS spectra for compound **9** in positive ion mode showing the absence of residual Ag^+^ ion.


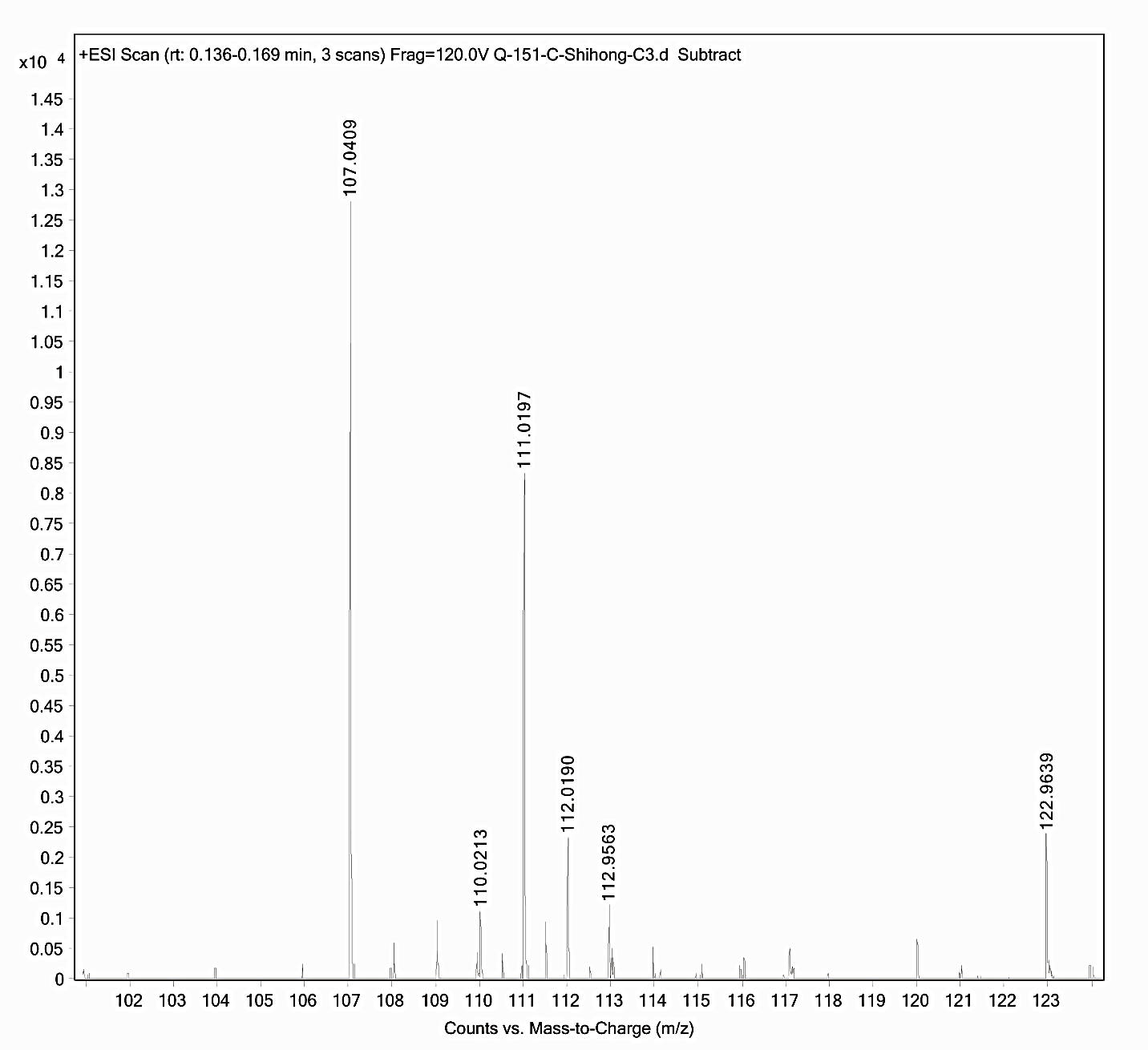


**Figure S12**. Low molecular weight regions of ESI-MS spectra for compound **10** in positive ion mode showing the absence of residual Ag^+^ ion.


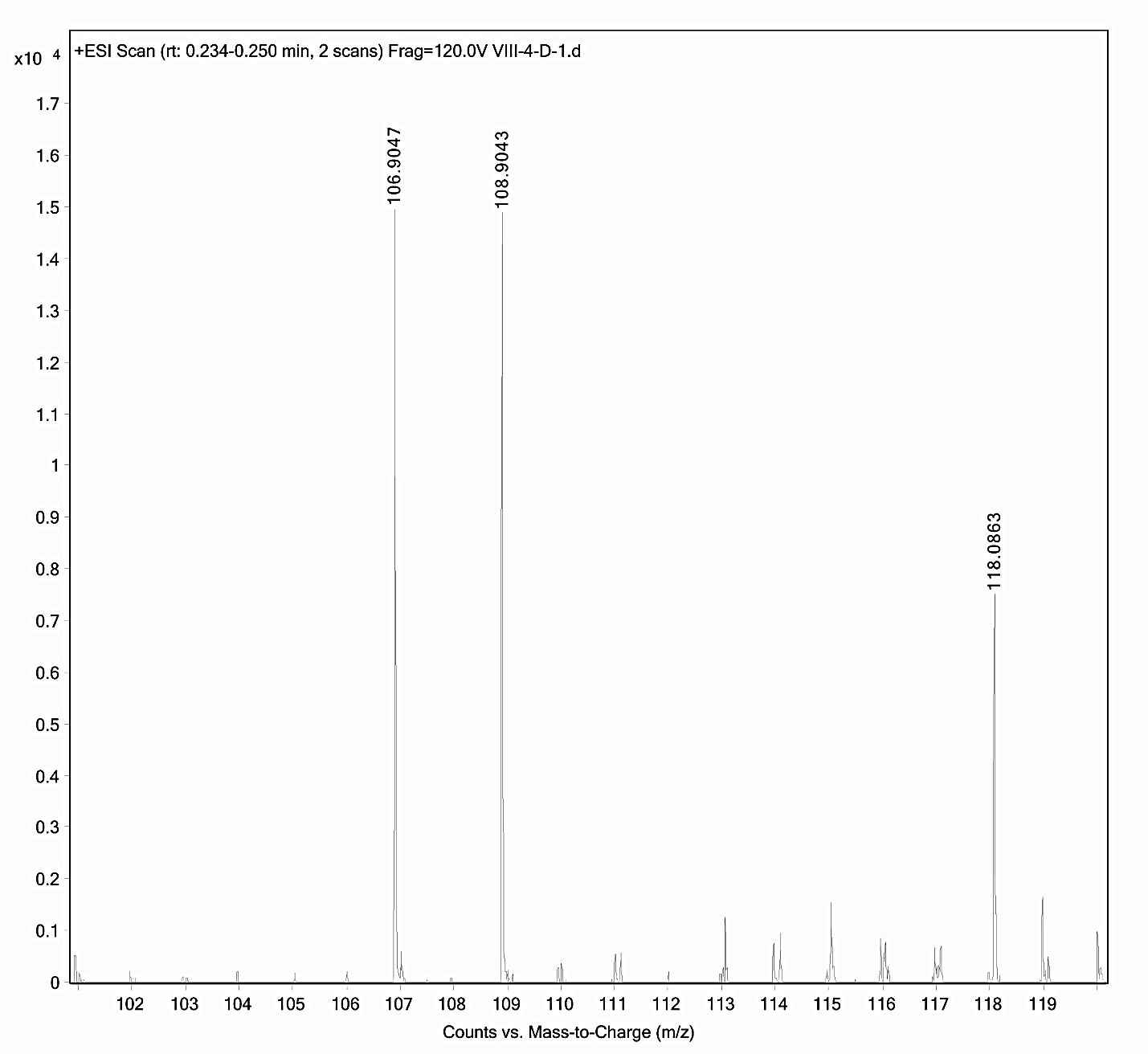


**Figure S13**. ESI-MS spectrum of control spectrum showing Ag^+^ signals at 106.9047 (100%) and 108.9043 (92.9%).
